# Left ventricular internal flow fraction from cardiac magnetic resonance images is higher in patients who respond to cardiac resynchronization therapy

**DOI:** 10.1186/1532-429X-14-S1-P216

**Published:** 2012-02-01

**Authors:** Stephanie Clement-Guinaudeau, Jonathan Suever, Antonello D'Andrea, Frits Prinzen, Michael Lloyd, Angel R Leon, John N Oshinski

**Affiliations:** 1 Emory University, Atlanta, GA, USA; 2 Georgia Institute of Technology, Atlanta, GA, USA; 3 Second University of Naples, Naples, Italy; 4 Maastricht University, Maastricht, Netherlands

## Summary

Despite the guidelines for patient selection for cardiac resynchronization therapy (CRT), one third of the patients do not benefit from the therapy. We show that internal flow fraction (IFF), an MRI-based dyssynchrony measure, was higher in responders to CRT than the non-responders. Therefore, IFF could be a useful to select patients who will response to CRT.

## Background

One third of the patients selected by the standard criteria to receive a cardiac resynchronization therapy (CRT) do not positively respond by clinical or functional outcome parameters. A large scar burden by late Gadolinium enhancement cardiac magnetic resonance (CMR) has been associated with non-response to CRT. However, CMR parameters, which predict response in non-ischemic patients by CMR are limited, or are complex to employ.

Internal Flow Fraction (IFF) is a novel parameter determined from cine images, which measures the fraction of flow over the cardiac cycle that sloshes between the walls of the LV (due to dyssynchronous contraction) compared to the blood that is ejected. It represents an image-based assessment of the amount of non-useful work (not contributing to ejection fraction) done during LV contraction. The purpose of this study was to evaluate whether IFF is higher in patients that positively response to CRT compared to non-responders to CRT.

## Methods

38 non-ischemic patients who met criteria for CRT were included in the study. Patients came from three centers (Emory University (Atlanta, USA), Maastricht University (Maastricht, NL), Second University of Naples (Naples, IT)). CMR was obtained pre-CRT on 1.5T MRI systems. Global IFF was determined from short-axis cine SSFP images using an in-house developed MATLAB program. Clinical response to CRT was assessed by the decrease in the NYHA class at 6-month follow-up.

## Results

**Figure 1:**
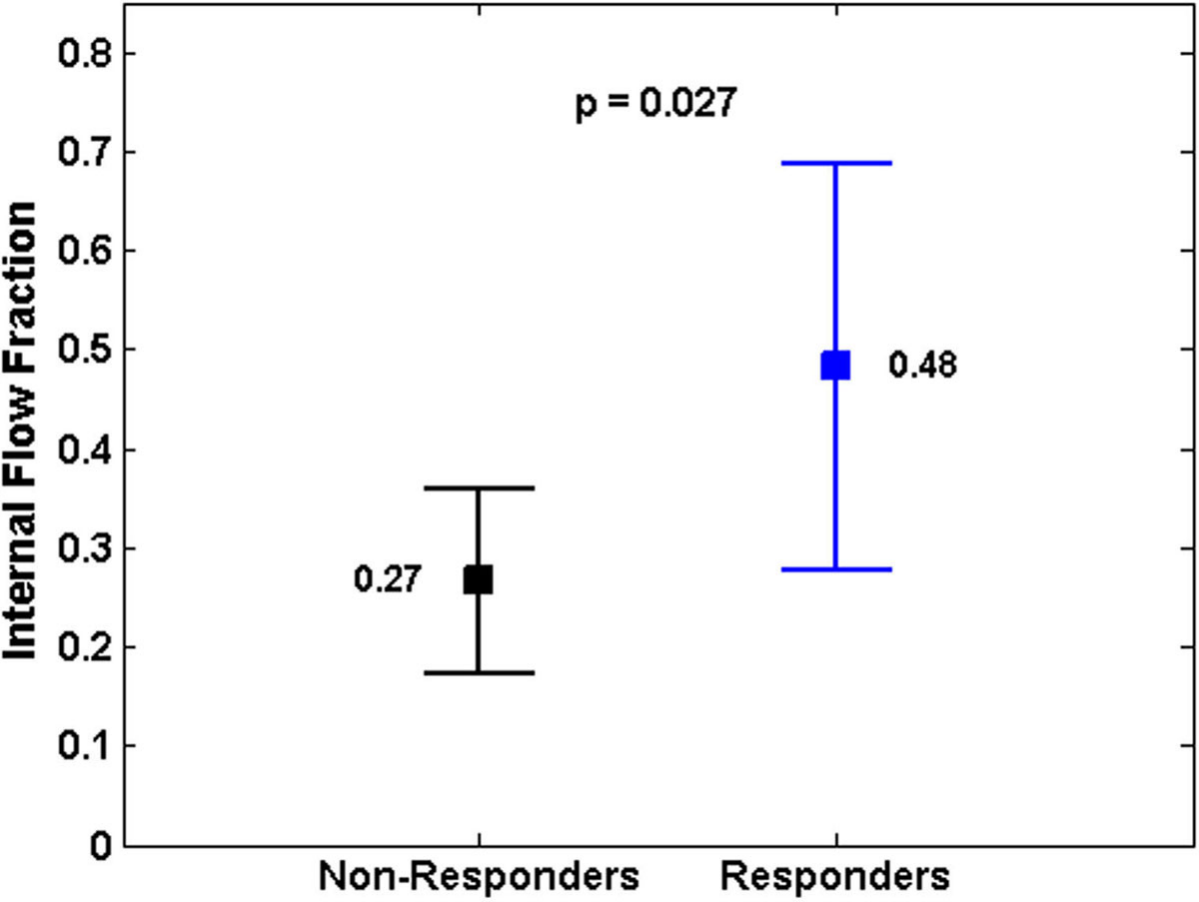


In our patient group, 66% of patients were positive responders. In the responders, the average IFF at baseline was significantly higher than in the non-responders group (0.26± 0.09 vs. 0.48± 0.2, p=0.027), Figure 1.

## Conclusions

IFF is higher in patients who respond positively to CRT than non responders. Therefore, IFF could be a useful tool to predict which patients will benefit from the CRT.

## Funding

This study was funded by a grant-in-aid from the American Heart Association.

